# Overexpression of lncRNA ATP11A‐AS1 in Colorectal Cancer and Its Association With *H. pylori* Infection

**DOI:** 10.1155/ijog/9351063

**Published:** 2026-07-13

**Authors:** Nima Hagholshahri, Nashwah Jabbar Kadhim Muttwaqi, Reza Safaralizadeh, Mohammadali Hosseinpour Feizi, Ali Rajabi

**Affiliations:** ^1^ Department of Animal Biology, Faculty of Natural Sciences, University of Tabriz, Tabriz, Iran, tabrizu.ac.ir; ^2^ Department of Forensic Science, College of Science, University of Al Qadisiyah, Al Diwaniyah, Iraq, qu.edu.iq

**Keywords:** ATP11A-AS1, colorectal cancer, *Helicobacter pylori*, long noncoding RNA, qRT-PCR

## Abstract

**Background:**

Colorectal cancer (CRC), the third most common cancer globally, is a leading cause of cancer‐related mortality. *Helicobacter pylori* infection, a risk factor for CRC, promotes carcinogenesis via inflammation and microbiota disruption. Long noncoding RNAs (lncRNAs), such as ATP11A‐AS1, regulate key processes in CRC progression, including proliferation and invasion. However, the role of ATP11A‐AS1 in CRC and its association with *H. pylori* infection remain underexplored. This study quantified ATP11A‐AS1 expression in CRC tumors compared with adjacent nontumor tissues and investigated its relationship with *H. pylori* infection and clinicopathological features.

**Methods:**

Total RNA was extracted from 100 paired tumor and adjacent nontumor tissues collected from CRC patients (53 males, 47 females, mean age: 56 ± 6.72). Following cDNA synthesis, ATP11A‐AS1 expression was measured via qRT‐PCR. Associations with clinicopathological factors (age, gender, tumor site, histology, stage, lymph node metastasis, *H. pylori* status, family history of CRC, alcohol consumption, and diabetes) were analyzed using Mann–Whitney tests. Multivariate binary logistic regression, adjusted for age, sex, and tumor site, was applied to clinicopathological outcomes.

**Results:**

*ATP11A-AS1* expression was significantly upregulated in tumor tissues compared with nontumor tissues (*p* < 0.0001). Among 59 *H. pylori*‐positive patients, expression was higher (*p* = 0.036). No significant associations were found with other features. Multivariate analysis confirmed no independent associations (all *p* > 0.05). ROC curve analysis yielded an AUC of 0.69, with sensitivity of 63% and specificity of 68%, indicating that *ATP11A-AS1* is a weak biomarker for CRC detection.

**Conclusion:**

ATP11A‐AS1 is overexpressed in CRC and associated with *H. pylori* infection, suggesting a potential role in CRC pathogenesis. Further studies are needed to elucidate its molecular mechanisms and clinical significance in *H. pylori*‐related CRC.

## 1. Introduction

Colorectal cancer (CRC), encompassing cancers of the colon and rectum, is the third most frequently diagnosed cancer globally, accounting for approximately 9.6% of cancer cases. It is also the second leading cause of cancer‐related deaths, responsible for 9.3% of mortalities worldwide. The incidence and mortality rates are notably higher in men than women, with rising early‐onset cases amplifying their global burden [1].

Despite advances in screening, particularly through colonoscopy, CRC remains a major global health challenge due to its often asymptomatic early stages, which delay diagnosis, lead to poorer prognosis, and limit effective treatment. The invasiveness and variable sensitivity of colonoscopy further hinder early detection, exacerbating CRC′s high mortality burden [2]. Although CRC incidence in individuals over 50 has stabilized in developed countries, the rising early‐onset CRC in younger populations highlights the need for deeper insights into its risk factors [3, 4]. Key risk factors for CRC include aging, male sex, dietary habits, genetic predisposition, and infections. For instance, *Helicobacter pylori* infection can alter the gut microbiota and impact gut immunity, thereby contributing to CRC incidence [5, 6]. In detail, *H. pylori* promote CRC through chronic inflammation via NF‐*κ*B activation, cytokine release (e.g., IL‐6), as well as gut microbiota dysbiosis [7]. Diabetes is another established risk factor, both increasing incidence and affecting prognosis through various dysregulated pathways. [8] As for tumor features, tumor progression is staged from I to IV based on factors such as tumor size, lymph node involvement, and metastases, with later stages posing increasing treatment challenges [9]. Moreover, approximately 10%–20% of CRC patients exhibit a mucinous subtype, characterized by abundant extracellular mucin compared with conventional adenocarcinoma [10]. Tumor microenvironment (TME) can also vary from case to case and include a wide range of bacteria, since TME can influence both internal and external conditions these influences can be case specific [11, 12]. These clinicopathological characteristics underscore the complexity of CRC and the need for molecular insights that could improve disease management.

Among emerging areas of molecular oncology, noncoding RNAs (ncRNAs) have garnered significant attention for their regulatory roles in cellular processes, despite lacking protein‐coding potential. Long noncoding RNAs (lncRNAs), defined as transcripts exceeding 200 nucleotides, are pivotal in modulating transcription, cell cycle progression, apoptosis, and genomic stability [13, 14]. Distributed across the genome, lncRNAs are categorized as intronic, intergenic, sense, or antisense based on their genomic origin. Transcribed primarily by RNA Polymerase II, most lncRNAs undergo capping, polyadenylation, and splicing, mirroring mRNA biogenesis, and are predominantly localized in the nucleus, with limited cytoplasmic presence [15]. Characterized by cell‐specific expression and low abundance, lncRNAs exert precise regulatory control in normal physiology. However, aberrant lncRNA expression is increasingly implicated in carcinogenesis, particularly in CRC, where it disrupts critical signaling pathways such as Wnt/*β*‐catenin and PI3K/AKT, alters protein expression, and promotes oncogenic phenotypes, including enhanced proliferation, migration, and invasion [16, 17]. For instance, lncRNAs like HOTAIR and MALAT1 have been shown to interact with chromatin‐modifying complexes or act as molecular sponges for microRNAs, thereby driving CRC progression. These roles in tumor initiation and progression underscore lncRNAs as key contributors to CRC pathogenesis and potential therapeutic targets [18].

ATP11A‐AS1, a lncRNA located on Chromosome 13, is transcribed antisense to the *ATP11A* gene, which encodes a phospholipid‐transporting ATPase involved in membrane lipid asymmetry and affecting innate immune response [19, 20]. Moreover, ATP11A overexpression serves as a metachronous metastasis predictor in CRC [21]. Emerging evidence indicates that ATP11A‐AS1 is significantly upregulated in colon adenocarcinoma and acute lymphoblastic leukemia, suggesting its oncogenic role in tumorigenesis. Despite these findings, the specific role of ATP11A‐AS1 in CRC pathogenesis remains largely unexplored [22, 23].

This study is aimed at quantifying ATP11A‐AS1 expression in CRC tissues compared with adjacent noncancerous tissues and to elucidate its associations with clinicopathological features, thereby providing insights into its potential as a biomarker or therapeutic target in CRC.

## 2. Material and Methods

### 2.1. Samples

One hundred tumor and tumor‐adjacent tissue samples were collected from CRC patients admitted to Imam Reza Hospital of Tabriz. The study included adult patients with a confirmed diagnosis of CRC. Patients were excluded if they had a concurrent or recent history of other primary malignancies or significant comorbid conditions. Additionally, individuals who had undergone prior treatment for CRC were not eligible for inclusion. Tissues were immediately placed in RNAse‐free microtubes, flash‐frozen in liquid nitrogen, and stored at −80°C until RNA extraction. This study was approved by the medical ethics committee of the University of Tabriz (ID No. IR.TABRIZU.REC.1403.113) and adhered to the Declaration of Helsinki and Good Clinical Practice guidelines. Written informed consent was obtained from all participants. An experienced pathologist confirmed the histological diagnosis for all samples.

### 2.2. RNA Extraction, Complementary DNA (cDNA) Synthesis, and Quantitative Real‐Time Polymerase Chain Reaction (qRT‐PCR)

Total RNA was extracted from frozen CRC tumor and paired adjacent nontumor tissues using TRIzol Reagent (Invitrogen, Thermo Fisher Scientific, Waltham, Massachusetts, United States) following the manufacturer′s protocol. To eliminate genomic DNA contamination, RNA samples were treated with DNase I (GeneAll Biotechnology, Seoul, South Korea) according to the manufacturer′s instructions. RNA concentration and purity were quantified using a NanoDrop 2000 spectrophotometer (Thermo Fisher Scientific, Wilmington, Delaware, United States), with A260/A280 ratios between 1.8 and 2.0 indicating acceptable purity. RNA integrity was assessed via 1% (*w*/*v*) agarose gel electrophoresis, confirming intact 28S and 18S rRNA bands.

cDNA was synthesized from 500 ng of total RNA using the PrimeScript RT Reagent Kit (TaKaRa Bio, Kusatsu, Japan) per the manufacturer′s protocol, with reactions performed in a 20 *μ*L volume. qRT‐PCR was conducted using SYBR Green PCR Master Mix (Amplicon, Odense, Denmark) on a LightCycler 96 System (Roche Molecular Systems, Pleasanton, California, United States). Each 20 *μ*L reaction contained 10 *μ*L SYBR Green Master Mix, 0.3 *μ*M of each forward and reverse primer, 1 *μ*L cDNA (100 ng/*μ*L) template, and nuclease‐free water. The primers for ATP11A‐AS1 were: forward, 5 ^′^‐CCACAAGTGCAGCGTTCATC‐3 ^′^; reverse, 5 ^′^‐AAGTGGTACTGACGTGTGGC‐3 ^′^. *β*‐Actin, used as the reference gene for normalization, was amplified with: forward, 5 ^′^‐AGAGCTACGAGCTGCCTGAC‐3 ^′^; reverse, 5 ^′^‐AGCACTGTCTTGGCGTACAG‐3 ^′^. The qRT‐PCR cycling conditions were as follows: initial denaturation at 95°C for 10 min, followed by 40 cycles of 95°C for 30 s, 60°C for 30 s, and 72°C for 30 s. Relative expression of ATP11A‐AS1 was calculated using the 2 − *ΔΔ*Ct method, normalized to *β*‐actin. And all experiments were conducted in triplicate.

### 2.3. Statistical Analysis

Cycle threshold (Ct) values for ATP11A‐AS1 and *β*‐actin were determined, with *Δ*Ct calculated as their differences. Relative expression was then quantified using the 2^−*Δ*Ct^ method for comparison between tumor and adjacent nontumor tissues. Moreover, GraphPad Prism 10 software was used to analyze the data and determine the sensitivity percentage, specificity percentage, and cutoff score, using the Index of Union (IU) method, for ATP11A‐AS1 as a potential biomarker in CRC patients by employing the receiver operating characteristic (ROC) curve test. Positive likelihood ratio (LR+) and negative likelihood ratio (LR−) were calculated using sensitivity/1‐specificity and 1‐sensitivity/specificity, respectively. Data normality was evaluated using both the Shapiro–Wilk and Kolmogorov–Smirnov tests. Continuous variables were summarized as mean ± standard deviation (SD). Associations between ATP11A‐AS1 expression level and the clinicopathological features were also evaluated using SPSS 20, employing the Mann–Whitney test for two‐group comparisons and multivariate analysis to assess the combined effects of multiple clinicopathological variables. A *p* value less than 0.05 was considered statistically significant.

## 3. Results

One hundred CRC patients (53 males, 47 females) with a mean age of 56 (±SD 6.72) were included. Sixteen patients were under 50, whereas 84 were 50 or older. Tumors were located in the rectum for 61 patients and in the colon for the remaining 39. Among the patients, 66 had adenocarcinoma, whereas 34 had mucinous adenocarcinoma. Tumor differentiation was poor in 42 cases, with the remaining exhibiting moderate or better differentiation. Early‐stage tumors (I/II) were observed in 52 cases, whereas 48 patients were diagnosed at advanced stages (III/IV). Lymph node metastasis was present in 59 cases, whereas 41 showed no metastasis. *H. pylori* was detected in 59 patients. A family history of CRC was reported in 46% of the patients. Additionally, 47 patients were diagnosed with diabetes, and 38 reported alcohol consumption.

### 3.1. Data Normality, Expression Levels, and Diagnostic Biomarker Potency

The Shapiro–Wilk test produced W values of 0.6507 and 0.6307 for tumoral and marginal samples, respectively, with *p* < 0.0001, indicating departure from a normal distribution. The Kolmogorov–Smirnov test yielded KS distances of 0.2406 and 0.2433 for tumor and marginal samples, also with *p* < 0.0001. Both tests showed that the datasets did not follow a normal distribution.

**Figure 1:**
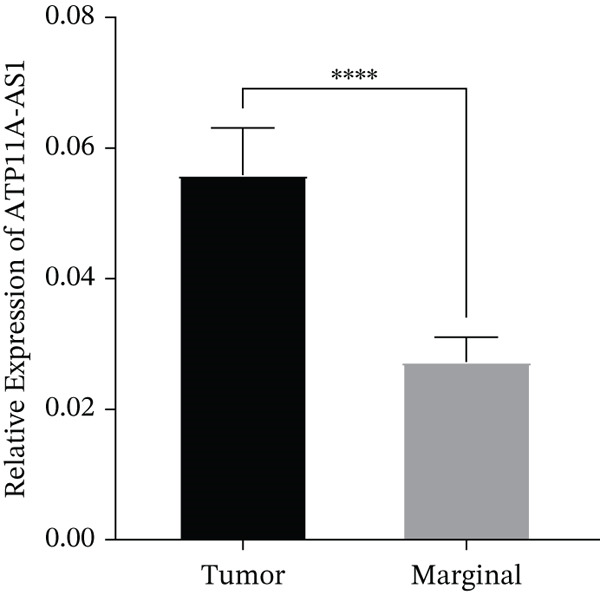
Relative expression of *ATP11A-AS1* in CRC tumor tissues compared with marginal nontumor tissues. Tumor tissues showed significantly higher *ATP11A-AS1* expression than marginal tissues (*p* < 0.0001), indicating potential upregulation in CRC. Bars represent mean ± SD; ****p < 0.0001.

The expression of ATP11A‐AS1 lncRNA was significantly higher in the tumor tissue compared with the adjacent noncancer tissue (*p* < 0.0001) (Figure 1). In *H. pylori*‐positive samples, ATP11A‐AS1 expression was elevated (*p* = 0.036). Other clinicopathological features did not correlate with ATP11A‐AS1 expression (Table 1). Multivariate binary logistic regression models, adjusted for age, sex, and tumor site, were fitted for all binary clinicopathological outcomes to evaluate the independent association of ATP11A‐AS1 tumor expression with these features (Table 2). Across the 10 outcomes, ATP11A‐AS1 expression showed no independent associations (all *p* > 0.05). Odds ratios (ORs) per unit increase in expression spanned a wide range (0.008–55.0) but remained consistently noninformative, reflecting the low variability in expression (SD = 0.072). Model fit was generally poor (McFadden′s Pseudo *R* ^2^ = 0.010–0.038 for converged models; LLR *p* > 0.14), indicating limited predictive power. Quasi‐complete or perfect separation occurred in 30% of models (e.g., diabetes and tumor site), yielding unstable estimates with confidence intervals spanning orders of magnitude (e.g., 0.0002–0.302 for diabetes; 0.000–∞ for tumor site), attributable to sparse event rates (*n* = 21–24 for diabetes, mucinous histology). The sex (male) model failed to converge due to collinearity. Notable but nonrobust trends included a borderline positive association with *H. pylori* infection (OR = 55.0, *p* = 0.184) and apparent protective effects for diabetes (OR = 0.008, *p* = 0.185) and poor differentiation (OR = 0.172, *p* = 0.558). Sex emerged as a confounder in the poor differentiation model (OR = 0.401, *p* = 0.030), with males less likely to exhibit poor differentiation.

**Table 1:** Association between ATP11A‐AS1 expression and clinicopathological features in patients with CRC.

Variable	Expression means in tumoral tissue (*m* *e* *a* *n* ± *S* *D*)	Expression means in marginal tissue (*m* *e* *a* *n* ± *S* *D*)	*p*
Age			0.067
< 50	0.096 ± 0.120	0.026 ± 0.031	
≥ 50	0.048 ± 0.055	0.028 ± 0.039	
Gender			0.111
Male	0.047 ± 0.056	0.033 ± 0.047	
Female	0.066 ± 0.086	0.021 ± 0.023	
Tumor site			0.677
Rectum	0.051 ± 0.058	0.025 ± 0.032	
Colon	0.063 ± 0.089	0.031 ± 0.045	
Tumor histology			0.483
Adenocarcinoma	0.057 ± 0.071	0.026 ± 0.030	
Mucinous adenocarcinoma	0.054 ± 0.074	0.030 ± 0.049	
Tumor differentiation			0.525
Poor	0.053 ± 0.081	0.028 ± 0.046	
Moderate/well	0.057 ± 0.064	0.026 ± 0.031	
TNM stages			0.738
I/II	0.054 ± 0.064	0.023 ± 0.031	
III/IV	0.057 ± 0.080	0.031 ± 0.046	
Lymph metastasis			0.866
No	0.055 ± 0.065	0.026 ± 0.040	
Yes	0.057 ± 0.081	0.028 ± 0.036	
*H. pylori* infection			0.036 ^∗^
No	0.048 ± 0.062	0.028 ± 0.045	
Yes	0.066 ± 0.083	0.026 ± 0.024	
Family history of CRC			0.161
No	0.054 ± 0.079	0.058 ± 0.062	
Yes	0.033 ± 0.048	0.020 ± 0.018	
Diabetes			0.462
No	0.067 ± 0.087	0.027 ± 0.028	
Yes	0.044 ± 0.047	0.028 ± 0.046	
Alcohol consumption			0.840
No	0.052 ± 0.058	0.030 ± 0.045	
Yes	0.063 ± 0.090	0.023 ± 0.023	

*p < 0.05.

**Table 2:** Summary of multivariate associations: tumor versus expression across all binary outcomes.

Outcome	Yes (*n*)	OR (95% CI)	*p*	Pseudo *R* ^2^	LLR *p* value
Tumor site (colon)	47	6.80 (0.000–∞)	1.000	1.000	1.000
Mucinous histology	21	0.410 (0.001–148.5)	0.773	0.012	0.190
Poor differentiation	28	0.172 (0.002–13.8)	0.558	0.037	0.711
Advanced TNM stage (III, IV)	48	2.16 (0.007–636.1)	0.791	0.010	0.158
Lymph metastasis	41	1.015 (0.006–173.4)	0.996	0.019	0.360
*H. pylori* infection	35	55.0 (0.150–20,200)	0.184	0.015	0.280
Family history of CRC	41	3.27 (0.011–972.5)	0.683	0.010	0.141
Diabetes	24	0.008 (0.0002–0.302)	0.185	0.038	0.738
Alcohol consumption	32	3.28 (0.011–980.5)	0.688	0.026	0.522

**Figure 2:**
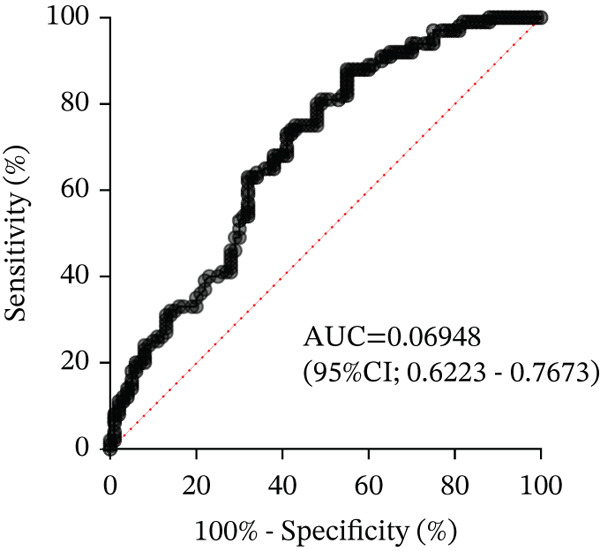
Receiver operating characteristic (ROC) curve illustrating the diagnostic performance of *ATP11A-AS1* expression in distinguishing colorectal cancer tissues from adjacent nontumor tissues. AUC was 0.6948 (95% CI: 0.6223–0.7673), indicating moderate discriminative ability. At the optimal cutoff, determined sensitivity was 63% and specificity was 68%.

To determine the biomarker potency of ATP11A‐AS1, the sensitivity and specificity were evaluated using the ROC curve analysis. Using the cutoff value of 0.023, the sensitivity and specificity of ATP11A‐AS1 were 63% and 68%, respectively (Figure 2). The area under the curve (AUC) was equal to 0.6948, which implies ATP11A‐AS1 is a weak biomarker. As for likelihood ratios, LR+ was equal to 1.97 (< 2) and LR− was equal to 0.54 (> 0.5), both of which showed a limited predictive power.

## 4. Discussion

CRC is the second most lethal type of cancer, demanding immediate research and clinical intervention. Its incidence is driven by an interplay of environmental and genetic factors [7]. Moreover, CRC is a heterogeneous disease that can be categorized into distinct subgroups based on tumor location (colon or rectum), stage (I–IV), histological features (mucinous or nonmucinous), and differing molecular profiles [2]. Given the asymptomatic nature of this cancer, it is commonly detected in its advanced stages. Late diagnosis complicates treatment and increases the risk of metastasis, which occurs in 30%–50% of patients [24]. Additionally, the primary diagnostic method, colonoscopy, is invasive and inaccurate [25]. As a result, there is a need for less invasive diagnostic markers and improved treatment strategies.

LncRNAs are a specific subgroup of ncRNAs that have significant roles in translation, apoptosis, and signaling, among other cellular functions [26]. Altered expression of lncRNAs can indicate various diseases such as cancer [27, 28]. For instance, the upregulation of PCAT‐1 [29] and MAFG‐AS1 [30] has been associated with CRC incidence. In addition to their correlation with cancer occurrence, lncRNAs may be linked to other cancer‐related characteristics, including cancer stage, metastasis probability, and histological features. As a result, expression patterns of lncRNAs can be used to distinguish various subtypes of a specific cancer.

Focusing on ATP11A‐AS1, a lncRNA with relatively limited prior research, our study confirmed its significant upregulation in CRC tumor tissue compared with adjacent noncancerous tissue (*p* < 0.0001), echoing observations in other malignancies such as lymphoblastic leukemia and adenocarcinoma [22, 23]. This result suggests a potential role for ATP11A‐AS1 in tumor progression. However, its diagnostic utility as a standalone biomarker is limited: ATP11A‐AS1 demonstrates an AUC of 0.69. These metrics indicate suboptimal performance in distinguishing CRC tissues from noncancerous ones.

Additionally, our investigation into clinicopathological factors revealed that among the variables examined, only *H. pylori* infection was significantly associated with elevated ATP11A‐AS1 expression (*p* = 0.036). This finding is consistent with the growing evidence linking gut microbiome imbalances, particularly bacterial infections, to CRC risk [31, 32]. Deeper understanding of the microbiome can help us use it as an early indicator of cancer [33]. In addition, bacterial characteristics of both TME and organ microbiome can influence cancer on many levels, including progression, metastasis, and treatment response [11, 34]. Due to their effects on cancer progression, these microbiome signatures can be utilized as biomarkers to predict cancer behavior [35]. Among the involved bacteria, *H. pylori* infection leads to higher CRC rate and mortality [5]. In particular, it has been implicated in CRC pathogenesis by altering the local immune environment and inflammatory response [6, 36]. Another mechanism, through which *H. pylori* makes the host vulnerable to CRC incidence and progression, is via altering the microbiota. These changes occur both in the gastric microbiota, the site of infection, and in the large intestine [37]. Thus, its eradication is associated with a reduced long‐term cancer risk despite a high annual incidence rate [38].

The present study did not investigate the molecular pathways through which *ATP11A-AS1* may contribute to CRC progression. Its interactions with other established biomarkers also remain unexplored, leaving uncertainty about whether *ATP11A-AS1* could serve as part of a biomarker panel despite its limited standalone diagnostic potency. In addition, the analysis was restricted to *H. pylori* infection, without considering the broader gut microbiota. Studying other bacterial communities could provide deeper insight into how *H. pylori* interacts with the microbial environment to influence *ATP11A-AS1* expression.

## 5. Conclusion

Overall, our findings underline the multifaceted nature of CRC and the complexities involved in its molecular diagnosis. Our study demonstrates a significant upregulation of ATP11A‐AS1 in CRC tissues compared with adjacent nontumor samples. We also observed a significant association between ATP11A‐AS1 expression levels and *H. pylori* infection. Although ATP11A‐AS1 is associated with CRC progression, its modest diagnostic performance suggests that it may be more valuable as part of a multibiomarker panel rather than as an individual indicator. Future research should focus on elucidating the molecular functions of ATP11A‐AS1 and its interactions with other RNAs, which could pave the way for integrated diagnostic strategies that enhance overall accuracy and improve patient outcomes.

## Author Contributions

**Nima Hagholshahri:** writing – original draft, visualization, software, methodology, conceptualization. **Nashwah Jabbar Kadhim Muttwaqi:** conceptualization, visualization, methodology, writing – original draft, software. **Reza Safaralizadeh:** writing – review & editing, visualization, supervision, conceptualization. **Mohammadali Hosseinpour Feizi:** writing – review & editing, and methodology. **Ali Rajabi:** conceptualization, methodology, formal analysis, writing – review & editing.

## Funding

No funding was received for this manuscript.

## Conflicts of Interest

The authors declare no conflicts of interest.

## Data Availability

The data that support the findings of this study are available from the corresponding authors upon reasonable request.
